# VAS2870 Inhibits Histamine-Induced Calcium Signaling and vWF Secretion in Human Umbilical Vein Endothelial Cells

**DOI:** 10.3390/cells8020196

**Published:** 2019-02-23

**Authors:** Pavel V. Avdonin, Elena Yu. Rybakova, Piotr P. Avdonin, Sergei K. Trufanov, Galina Yu. Mironova, Alexandra A. Tsitrina, Nikolay V. Goncharov

**Affiliations:** 1 Koltsov Institute of Developmental Biology, Moscow 119334, Russia; alenka3107@mail.ru (E.Y.R.); ppavdonin@gmail.com (P.P.A.); gad.91@inbox.ru (S.K.T.); wereshelen@gmail.com (G.Y.M.); sashulka.s@gmail.com (A.A.T.); 2 Sechenov Institute of Evolutional Institute of Evolutionary Physiology and Biochemistry, Saint Petersburg 194223, Russia; ngoncharov@gmail.com; 3 Research Institute of Hygiene, Occupational Pathology and Human Ecology, Leningrad Region 188663, Russia

**Keywords:** histamine, calcium, endothelial cells, NADPH-oxidase, VAS2870, von Willebrand factor, aorta, relaxation

## Abstract

In this study, we investigated the effects of NAD(P)H oxidase (NOX) inhibitor VAS2870 (3-benzyl-7-(2-benzoxazolyl)thio-1,2,3-triazolo[4,5-d]pyrimidine) on the histamine-induced elevation of free cytoplasmic calcium concentration ([Ca^2+^]_i_) and the secretion of von Willebrand factor (vWF) in human umbilical vein endothelial cells (HUVECs) and on relaxation of rat aorta in response to histamine. At 10 μM concentration, VAS2870 suppressed the [Ca^2+^]_i_ rise induced by histamine. Inhibition was not competitive, with IC50 3.64 and 3.22 μM at 1 and 100 μM concentrations of histamine, respectively. There was no inhibition of [Ca^2+^]_i_ elevation by VAS2870 in HUVECs in response to the agonist of type 1 protease-activated receptor SFLLRN. VAS2870 attenuated histamine-induced secretion of vWF and did not inhibit basal secretion. VAS2870 did not change the degree of histamine-induced relaxation of rat aortic rings constricted by norepinephrine. We suggest that NOX inhibitors might be used as a tool for preventing thrombosis induced by histamine release from mast cells without affecting vasorelaxation.

## 1. Introduction

Histamine plays an important role as chemical mediator in multiple physiological and pathophysiological processes in central and peripheral tissues. Mast cells and basophils are important sources of histamine, which is released from granule stores in response to several stimuli. The pleiotropic effects of histamine are mediated via four G protein-coupled receptor (GPCR) subtypes (H_1_R–H_4_R), which differ in their distribution, ligand binding, signaling pathways, and functions [1]. Histamine acts as a full agonist on the receptors, with subtype-specific differences in affinity. H_1_R is ubiquitously expressed and mediates its effects by G_q/11_ activation via phospholipase C (PLC) and to a minor degree by PLA_2_ and PLD. Activation of H_1_R leads to an increase of inositol-1,4,5-triphosphate (IP_3_) and 1,2-diacylglycerol (DAG), associated with an increase of the intracellular Ca^2+^ concentration, followed by activation of protein kinase C (PKC). Other signaling pathways include the stimulation of adenylyl cyclase via H_2_R with the formation of cAMP, inducing the generation of NO; the NF-κB cascade leads to a release or increase in the expression of (pro)inflammatory mediators (P-selectin, ICAM-1, VCAM-1, iNOS, IL-1beta, IL-6, TNF-alpha, etc.) [2]. Brain vascular endothelial cells (ECs) express histamine H1 and H2 receptors, which regulate brain capillary permeability. Also, histamine receptor genes Hrh3 and Hrh4, for corresponding H3 and H4 receptors, are expressed in rat brain ECs, which are potentially important for the regulation of blood–brain barrier permeability, including trafficking of immunocompetent cells [3]. H4 receptors play a dominant role in histamine-induced eosinophil adhesion to endothelium [4].

Reactive oxygen species (ROS) in large amounts clearly have detrimental effects on cell physiology, whereas low concentrations of ROS are permanently produced in cells and play a role as signaling molecules. An imbalance in ROS production and defense mechanisms can lead to pathological vascular remodeling. Among the possible sources of ROS within endothelial and/or neighboring cells are mitochondria, NADPH-oxidases, xanthine oxidase, peroxidases, NO-synthases, cytochrome P450, cyclooxygenases, lipoxygenases, monoamine oxidases, and the hemoglobin of red blood cells [5,6,7]. Among these possible sources of ROS, nicotinamide adenine dinucleotide phosphate (NADPH) oxidases (NOXs) play a central role. NOXs are present in neutrophils, where they mostly determine the non-specific immune response [8]. In the ischemic-reperfusion condition, NOXs substantially determine tissue injury by generating ROS in ECs and smooth muscle cells (SMCs) of blood vessels [9]. 

In ECs, four NOX isoforms include the superoxide-generating enzymes NOX1, NOX2, and NOX5 and the hydrogen-peroxide-generating enzyme NOX4 [10]. NOX2 is the most studied isoform of NOX, which generates superoxide anion in professional phagocytes and many other cells. In ECs, it is the predominant form, and all four subunits of NOX2 are present [11,12]. It is activated by angiotensin II [13], oxidized low-density lipoproteins [14], cytokines, and growth factors [15]. Superoxide anion generated by NOX2 causes the inactivation of nitric oxide and promotes the development of endothelial dysfunction, hypertension, and inflammation [16]. NOX4 predominantly generates hydrogen peroxide (H_2_O_2_). H_2_O_2_ produced by endothelial NOX4 potentiates vasorelaxation induced by acetylcholine and histamine [17]. Activators of other NOXs do not affect the activity of NOX4, which is constitutive, since the generation of H_2_O_2_ in cells is dependent on the level of expression of NOX4 [18]. NADPH oxidases of the DUOX group (DUOX1 and DUOX2) also generate H_2_O_2_ [19,20], though their existence and/or functional significance in ECs and SMCs has not been clearly shown.

Calcium-dependent NOX5 has been implicated in the oxidative damage of ECs in atherosclerosis and hypertension [21]. It is noteworthy that NOX5 is expressed in primates but does not occur naturally in rodents. In transgenic mice expressing human NOX5 in a vascular SMC-specific manner, agonist-induced vasoconstriction was exaggerated, and endothelium-dependent vasorelaxation was attenuated [22].

The influence of ROS on Ca^2+^ signaling in EC has been demonstrated in several works [23,24,25,26,27,28,29,30]. ROS can activate various calcium channels of the endoplasmic reticulum and plasma membrane, such as InsP3- and ryanodine-sensitive channels, and some cation channels of the TRP superfamily [25,31,32]. NOX-derived ROS are critical for the generation of Ca^2+^ oscillations in response to histamine in human aortic endothelial cells [33]. Recently, we demonstrated an involvement of two-pore channels in an H_2_O_2_-induced increase in the level of calcium ions in the cytoplasm of human umbilical vein endothelial cells (HUVECs) [34]. This could be coupled with exocytosis of the von Willebrand factor (vWF) in these cells in response to H_2_O_2_ [35]. The vWF release from EC is induced by superoxide anion [30]. In the present work, we studied the role of NOX in histamine-induced Ca^2+^ rise and vWF secretion in HUVECs and NOX involvement in rat aorta relaxation in response to histamine. To solve these questions, we used VAS2870 as a tool inhibitor of NOXs [36]. VAS2870 belongs to the triazolopyrimidines, which are regarded as the most specific inhibitors of NOXs [37]. 

## 2. Methods

### 2.1. Reagents

VAS2870, histamine, and SFLLRN were from SigmaAldrich (St. Louis, MO, USA); CalciumGreen/AM and dihydroethidium were from Thermo Fischer Scientific (Waltham, MA, USA). VAS2870 was dissolved at a 10 mM concentration in DMSO. Before being added to the cells, it was dissolved to the required concentrations in physiological salt solution (PSS). DMSO at appropriate concentrations was used as a vehicle control. PSS was added as a vehicle control for histamine and SFLLRN.

### 2.2. Cell Culture

Human umbilical vein endothelial cells (HUVECs) were isolated according to [38]. The cells were grown in plastic dishes pre-coated with gelatin, using M199 medium with Earl’s salts and 20 mM HEPES containing 20% fetal calf serum (SigmaAldrich), 300 µg/mL endothelial growth supplement, isolated from rabbit brain, 100 µg/mL heparin and gentamicin. We used the cells on early passages (1–4). Accutase^®^ was applied for passaging the cells (SigmaAldrich).

### 2.3. Measurement of Free Cytoplasmic Calcium Concentration in HUVECs

HUVECs grown in 96-well plates were loaded with 1 µM CalciumGreen/AM dissolved with 0.02% Pluronic F-127 in M199 during 1 h at 37 °C in a CO_2_ incubator (New Brunswick Scientific, Edison, NJ, USA). Measurement of [Ca^2+^]_i_ was performed in physiological salt solution (PSS) containing NaCl (145 mM), KCl (5 mM), MgCl_2_ (1 mM), CaCl_2_ (1 mM), HEPES (5 mM), and D-glucose (10 mM), at pH 7.4. Fluorescence was registered at 485 nm (excitation) and 530 nm (emission) at 25 °C using a Synergy 4 Microplate Reader (BioTek, Winooski, VT, USA). The changes in [Ca^2+^]_i_ in HUVECs are presented as the ratio of the increment in F_530_ and initial F_530_. Each curve in in the graph is a superposition of three curves recorded simultaneously from three wells in a plate.

### 2.4. Registration of ROS Generation in HUVECs

Kinetics of ROS generation in HUVECs was registered with the fluorescent indicator dihydroethidium. The fluorescence was measured by a Synergy 4 Microplate Reader (BioTek) with excitation filter 485 nm (bandwidth 20 nm) and emission 620 nm (bandwidth 40 nm). The bandwidth of the emission filter is enough wide to cover a large part of the emission of the oxidation products of DHE [39]. The cells grown in 96-well plates were incubated with different concentrations of VAS2870 or vehicle control (dimethylsulfoxide at final concentrations from 0.006% to 0.2%) during 5 min and then DHE at final concentration 2.5 μM was added. The slight inhibitory effect of DMSO on DHE oxidation at a concentration of 0.2% was taken into account when analyzing the data. The oxidation kinetics of DHE determined by the increase in fluorescence was linear during the 60 min of incubation both in the absence and in presence of VAS2870. The increase in fluorescence in the absence of VAS2870 was taken as 100%. The results are presented as the mean values obtained in three experiments with different cell preparations. In each experiment, the experimental point was a mean value of the fluorescence from six wells in a 96-well plate.

### 2.5. Measurement of vWF Secretion

HUVECs grown in 48-well plates were incubated in PSS at 30 °C during 5 min with or without 10 μM VAS2870. Then, histamine at final concentration or vehicle were added and the cells were incubated for 30 min. The extracellular fluid from each well was collected in a separate tube and frozen. Later, it was used for the measurement of vWF concentration with a TECHNOZYM vWF:Ag ELISA kit (Technoclone, Vienna, Austria). The values of the secreted vWF are the means of six measurements.

### 2.6. Registration of Aorta Contraction

Aorta were isolated from male Wistar rats weighing 250–300 g. The rats were anesthetized with 25% urethane (4 mL/kg) and decapitated. All manipulations with the animals were performed in accordance with the guide for the care and use of Laboratory animals of the Bioethics committee of the Koltsov Institute of Developmental Biology and European Convention for the Protection of Vertebrate Animals used for Experimental and Other Scientific Purposes. Aorta were cleaned from connective tissue and cut into rings with a width of around 2 mm. Experiments were performed on a four-channel wire myograph (ADInstruments, Bella Vista, New South Wales, Australia) using LabChart 7.3.7 program for data acquisition and analysis. The rings were mounted on the holders in chambers filled with Krebs–Henseleit solution (37°C) perfused with 95% O_2_/5% СO_2_ and extended with a force of 1 g. Contractility of the vessel rings and intactness of its endothelium were tested by adding 10^−7^ M norepinephrine and then 10^−5^ M carbachol. After washing, norepinephrine and carbachol were added again. Aortic rings with relaxation of at least 50% were used for the experiments.

### 2.7. Statistics

Data are presented as mean ± SEM. The number of measurements is presented in the legend to the figures. In each case at least three independent experiments were performed with different cell or vessel preparations. Statistical significance was calculated using Excel 2003 and MedCalc 18.9.1 statistical software according the Student-Neuman-Keuls test. The IC50 values were determined with GraphPad Prism 8.0.

## 3. Results and Discussion

**Figure 1:**
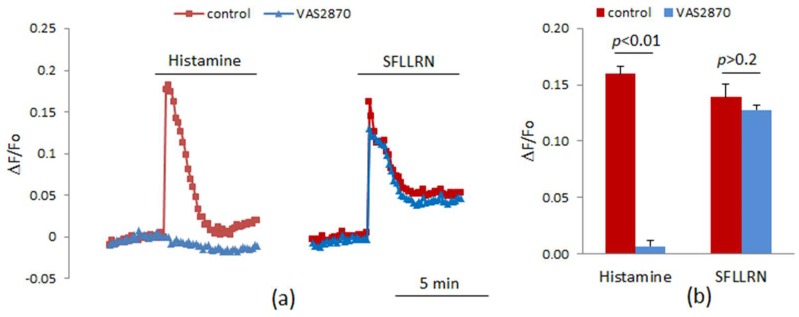
The influence of VAS2870 (10 µM) on calcium signaling in response to histamine (10 μM) and agonist of PAR1 SFLLRN (2 µg/mL). (**a**) The curves represent the data of one of three experiments with similar results; (**b**) The bars represent the mean values of [Ca^2+^]_i_ increase in three independent experiments. [Ca^2+^]_i_ was measured as a relative increase in CalciumGreen fluorescence. In control experiments, the vehicle, dimethylsulfoxide (DMSO) at a final concentration 0.1%, was added instead of VAS2870.

**Figure 2:**
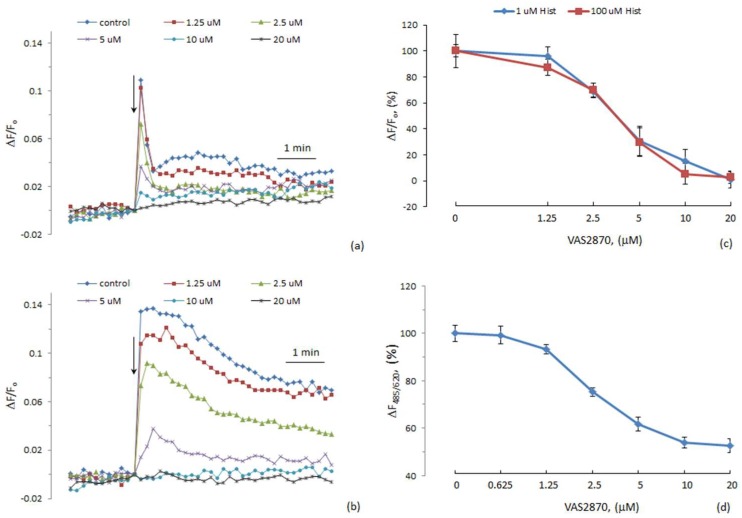
The influence of different concentrations of VAS2870 on histamine-induced [Ca^2+^]_i_ increases and on the generation of reactive oxygen species (ROS) in human umbilical vein endothelial cells (HUVECs). (**a**,**b**) The kinetics of [Ca^2+^]_i_ elevation in HUVECs in response to 1 and 100 μM histamine, respectively. (**c**) Concentration-dependent curves of the inhibition of [Ca^2+^]_i_ increases. (**d**) Concentration-dependence curve of the inhibition of dihydroethidium (DHE) oxidation by VAS2870. Each point in (**c**,**d**) is a mean of three values from three independent experiments on different cell preparations. In control experiments, it was demonstrated that DMSO at concentrations on which it was applied to the cells along with VAS2870 did not suppress histamine-induced [Ca^2+^]_i_ elevation and oxidation of DHE.

We studied the effect of VAS2870 on [Ca^2+^]_i_ elevation induced by histamine. At a concentration of 10 μM, VAS2870 almost completely suppressed the calcium signal of 1 mM histamine, while there was no significant inhibition of the action of PAR1 agonist SFLLRN (Figure 1). The effect of VAS2870 was not competitive toward histamine, since wat is observed at a low concentration of VAS2870, which is two orders of magnitude lower than the concentration of histamine. We determined the concentration-dependence curves for the inhibition by VAS2870 of [Ca^2+^]_i_ elevation at different histamine concentrations (Figure 2c). The IC50 for VAS2870 calculated from these data was 3.64 and 3.22 μM at 1 and 100 μM concentrations of histamine, respectively. These results suggest the existence of a specific mechanism of histamine-induced [Ca^2+^]_i_ mobilization in HUVECs. The following data provide evidence in favor of this conclusion. It has been shown that two-pore channels activated by NAADP are involved in histamine-induced [Ca^2+^]_i_ mobilization in HUVEC via H1 receptors, while calcium signaling of thrombin is independent from NAADP [40,41].

VAS2870 in micromolar concentrations is widely used to suppress NOX activity in different types of cells [42,43,44,45]. The IC50 for VAS2870’s effect on NOX activity in a cell-free system with membranes and cytosol from human neutrophils was 10.6 μM, and in intact HL-60 cells the IC50 was 2 μM [43]. However, according to Gatto et al. [46], in neutrophils, ROS generation is inhibited by VAS2870 at submicromolar concentrations with an IC50 of 77 nM. We determined which concentration of VAS2870 suppresses ROS production in HUVECs. For this purpose, dihydroethidium (DHE) was used. VAS2870 reduced the rate of DHE oxidation in HUVECs with an IC50 of 2.47 μM (Figure 2d). At a 20 μM concentration, VAS2870 suppressed nearly half of ROS formation measured with DHE, and its effect reached a plateau. The reason for this might be that oxidation of DHE in cells is caused only partially by superoxide anion [39]. The values of IC50 for the inhibition of ROS generation and suppression of histamine-induced [Ca^2+^]_i_ elevation in HUVECs were very close, which suggests a link between these processes. VAS2870 is an inhibitor of NADPH-oxidase isoforms NOX1/2/4 [47,48] and does not suppress NOX5 [22]. It has been demonstrated that VAS2870 reverses oxidative stress, which is caused by NOX1/2 activation [49]. We propose that the suppression of histamine-induced [Ca^2+^]_i_ increase in HUVECs might occur due to the inhibition of NOX2 because this form of NOX is under the control of several intracellular regulatory pathways [9], while the activity of NOX4 is mostly regulated by the level of expression. In addition, it has been shown that increased expression of NOX4 in endothelial cells enhances endothelium-dependent relaxation [17].

**Figure 3:**
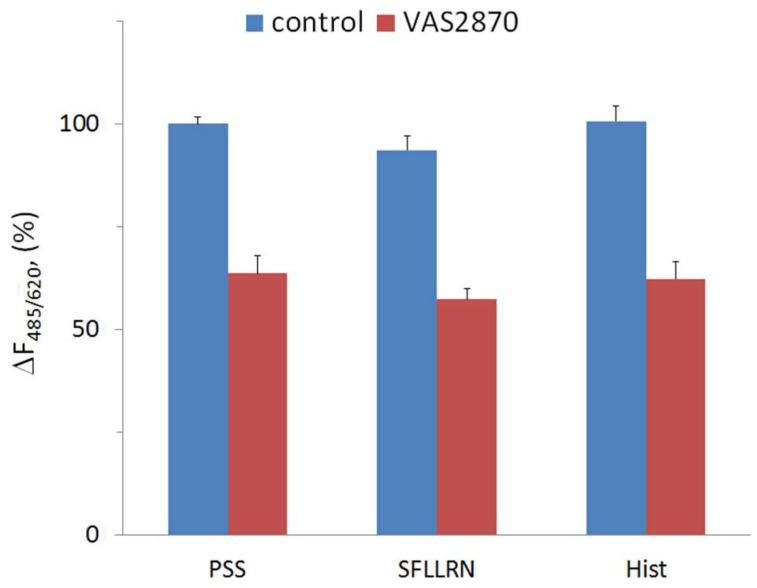
The influence of SFLLRN and histamine on DHE oxidation in the absence and presence of VAS2870. Physiological salt solution (PSS) was used as a vehicle control for the agonists. Each value is a mean of three values from three independent experiments with different cell preparations. DMSO at final concentration 0.1% was used as a vehicle control for VAS2870. The difference between the values in the absence and presence of VAS2870 was statistically significant (*p* < 0.01).

Evidence in favor of NOX involvement in histamine-induced calcium signaling was published quite a long time ago by Hu and co-workers [33], wherein they demonstrated that NOX-derived ROS were critical for generating the Ca^2+^ oscillations in response to histamine in human aortic ECs. Recently, it was demonstrated that in microglial cells, histamine stimulates ROS formation due to the activation of NOX1 via H1 receptors [50]. We expected that histamine might affect ROS generation in HUVECs. However, there was no increase in the rate of DHE oxidation in the presence of histamine (Figure 3). SFLLRN also did not produce any effect. It might be suggested that either histamine does not stimulate the generation of superoxide anion in HUVECs, or the method with DHE is not sensitive enough to detect the increment in ROS formation due to histamine. It should be mentioned that in [33] 2′,7′-dihydrodichlorofluorescin diacetate was used as a fluorescent probe. Elucidation of the mechanism of NOX involvement in signal transduction from histamine receptors in HUVECs requires further research.

**Figure 4:**
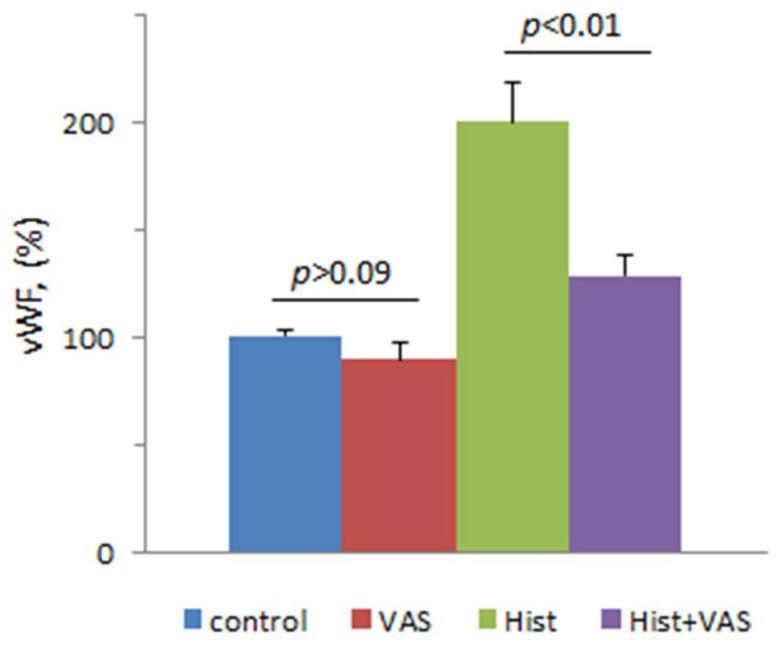
The influence of histamine on the secretion of von Willebrand factor (vWF) in the absence or presence of VAS2870. HUVECs were incubated at 30 °C during 5 min with 10 μM VAS2870 or vehicle (DMSO at final concentration 0.1% in the wells of a 48-well plate. Then, 100 μM histamine, or PSS as a vehicle control, were added to the cells, and they were additionally incubated for 30 min. Each value is a mean of the data from three independent experiments.

The next task was to study how VAS2870 can affect physiological reactions of ECs activated by histamine, which causes secretion of vWF in HUVECs due to the activation of H1 receptors. This secretion is mediated by the elevation of cytoplasmic calcium ion concentration [40,51]. We showed that, in the presence of VAS2870, the effect of histamine on vWF secretion was attenuated, though VAS2870 did not inhibit the basal secretion of vWF (Figure 4). This result correlates with our data on the inhibition of histamine’s effect on [Ca^2+^]_i_ by VAS2870.

**Figure 5:**
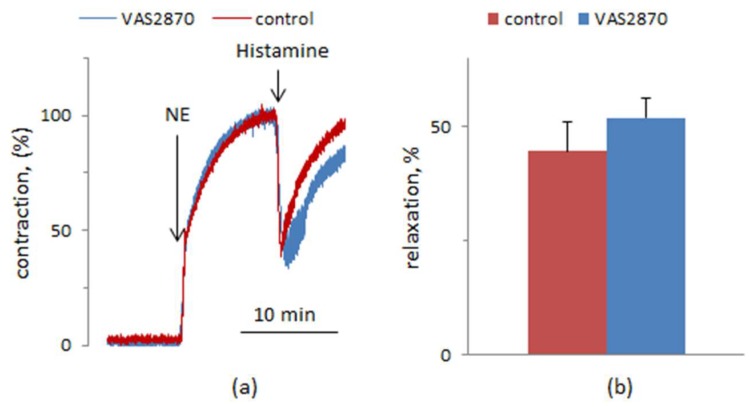
Relaxation of rat aortic rings preconstricted by norepinephrine (NE) in response to 100 μM histamine in the absence and presence of VAS2870. VAS2870 at a concentration of 10 μM or vehicle was added to the rings 30 min or 1 h before NE. (**a**) Typical curves of the contraction and relaxation. (**b**) The degrees of relaxation in the absence and presence of VAS2870 (*n* = 10 for control and for VAS2870 from five independent experiments). In each experiment there were measurements from two control and two experimental aortic rings.

Among the physiological effects of histamine, relaxation of blood vessels is one of the most important, so the next task was to evaluate the influence of VAS2870 on this function. We determined the histamine-induced decrease of the contraction force of rat aortic rings preconstricted with norepinephrine. The rings were preincubated for 30 or 60 min with VAS2870 or vehicle before adding norepinephrine, followed by the addition of histamine. At concentrations of 10 and 100 μM, histamine induced the relaxation of the rings. Surprisingly, there was no attenuation of histamine-induced relaxation after preincubation of the rings with VAS2870 (Figure 5). It can be assumed that in rat aortic ECs VAS2870 does not produce as strong an inhibition of histamine-induced [Ca^2+^]_i_ rise as in HUVECs. It is known that histamine elevates [Ca^2+^]i in HUVEC due to its mobilization from endoplasmic reticulum via the channels activated by inositol 1,4,5-trisphosphate and from endolysosomal vesicles via two-pore channels [40,41]. The relative roles of these two calcium signaling mechanisms might be different in ECs from rat aorta and human umbilical vein. Another possible explanation is that the role of calcium ions entering the cytoplasm from different sources is different in the case of vasorelaxation and vWF secretion. It has been demonstrated that the suppression of Ca^2+^ release from endolysosomal vesicles inhibits vWF secretion induced by histamine [40]. On the other hand, endothelium-dependent vasorelaxation depends on store-operated Ca^2+^ entry [52].

In [49], it was demonstrated that VAS2870 improves endothelium-dependent relaxation of spontaneously hypertensive rat (SHR) aortas induced by acetylcholine. The reason for the difference of these data and our results about the lack of VAS2870’s effect on endothelium-dependent vasorelaxation is the increased level of ROS in the aorta of SHR due to the elevated expression of NOX1 and NOX2. The inhibition of their activity by VAS2870 improved impaired the acetylcholine-induced relaxation of spontaneously hypertensive rat aortas [49]. In control normotensive Wistar-Kioto rats, VAS2870 does not affect endothelium-dependent relaxation. There is evidence that VAS2870 can normalize arteriolar flow-induced dilation caused by oxidative stress at hyperinsulinemia [53]. In experiments with murine aorta rings it was demonstrated that their incubation in conditions of hyperglycemia-induced oxidative stress causes impairment of vasodilation mediated by PAR2 agonists, and VAS2870 improved this function [54]. From all these data it can be concluded that VAS2870 is able to improve endothelial-dependent vasorelaxation at pathological state associated with oxidative stress when NOX1 and NOX2 activities are increased, while in normal vessels it does not affect relaxation. This is also supported by our data.

Inhibition of vWF secretion by VAS2870 indicates that ROS produced by NOXs are involved in this reaction. Direct activation of vWF secretion was demonstrated for superoxide anion [30]. We have recently shown that H_2_O_2_ induces the exocytosis of vWF in HUVECs [35]. The physiological significance of the stimulation of vWF exocytosis and secretion by ROS is not clear. However, this effect could take place in pathophysiological conditions when hyperactivation of vWF secretion occurs. It was demonstrated that histamine released from mast cells is one of the factors initiating deep vein thrombosis due to excessive secretion of vWF [55]. Thus, it could be suggested that NOX inhibitors might be used as a tool for preventing deep vein thrombosis induced by histamine release from mast cells without affecting vasorelaxation. In fact, the number of pathological states which could be targeted by VAS2870 or other NOX inhibitors together with or alternatively to other pharmaceuticals has been increasing over the past several years [56].

Endothelial activation initiates multiple processes, including hemostasis and inflammation. The molecules that contribute to these processes are co-stored in secretory granules, the Weibel-Palade bodies (WPBs) being the most known and important in ECs. It was previously shown that Ca^2+^-elevating agonists can deplete the cell of almost all WPBs, whereas cAMP-elevating agonists selectively release a pool of mature WPBs that contain vWF but little or no P-selectin [57]. NOX inhibitors can control the release of granule content from ECs to allow differentiated responses, and the development of selected agonists for tuning the endothelial response is currently an urgent problem [58].
